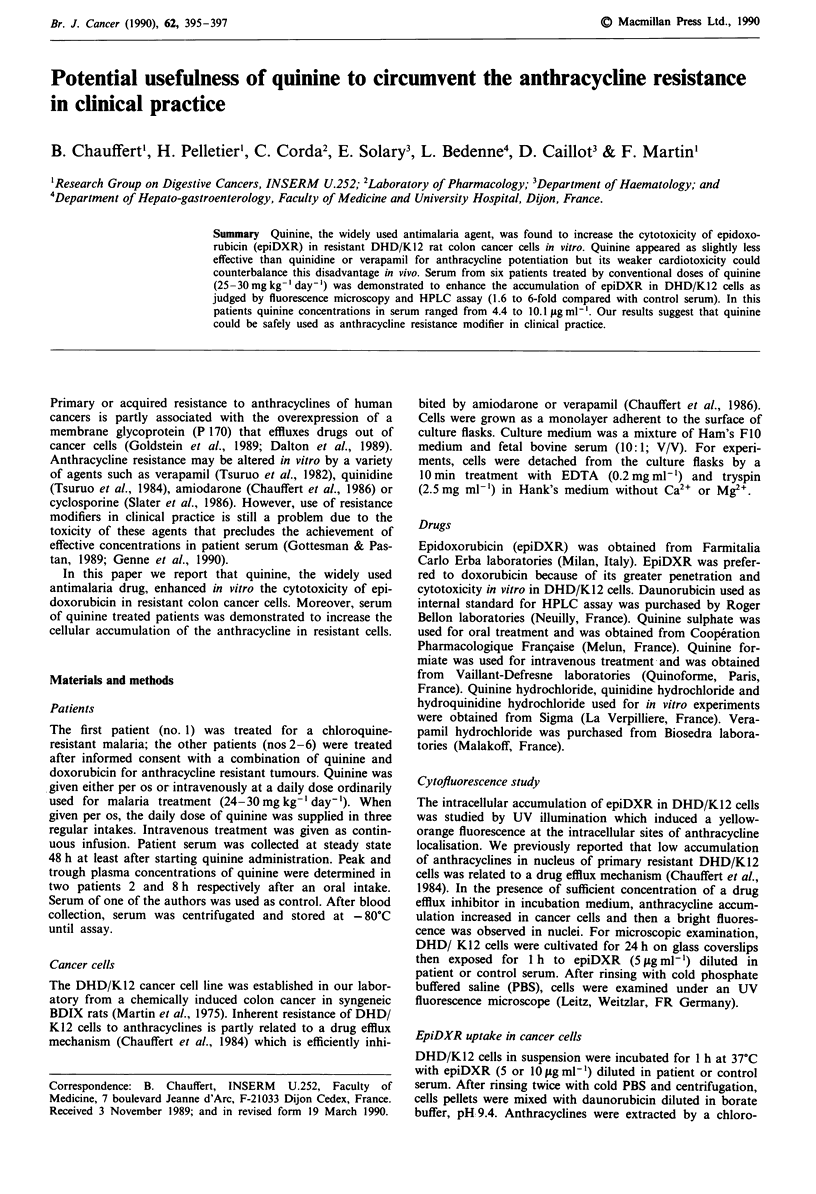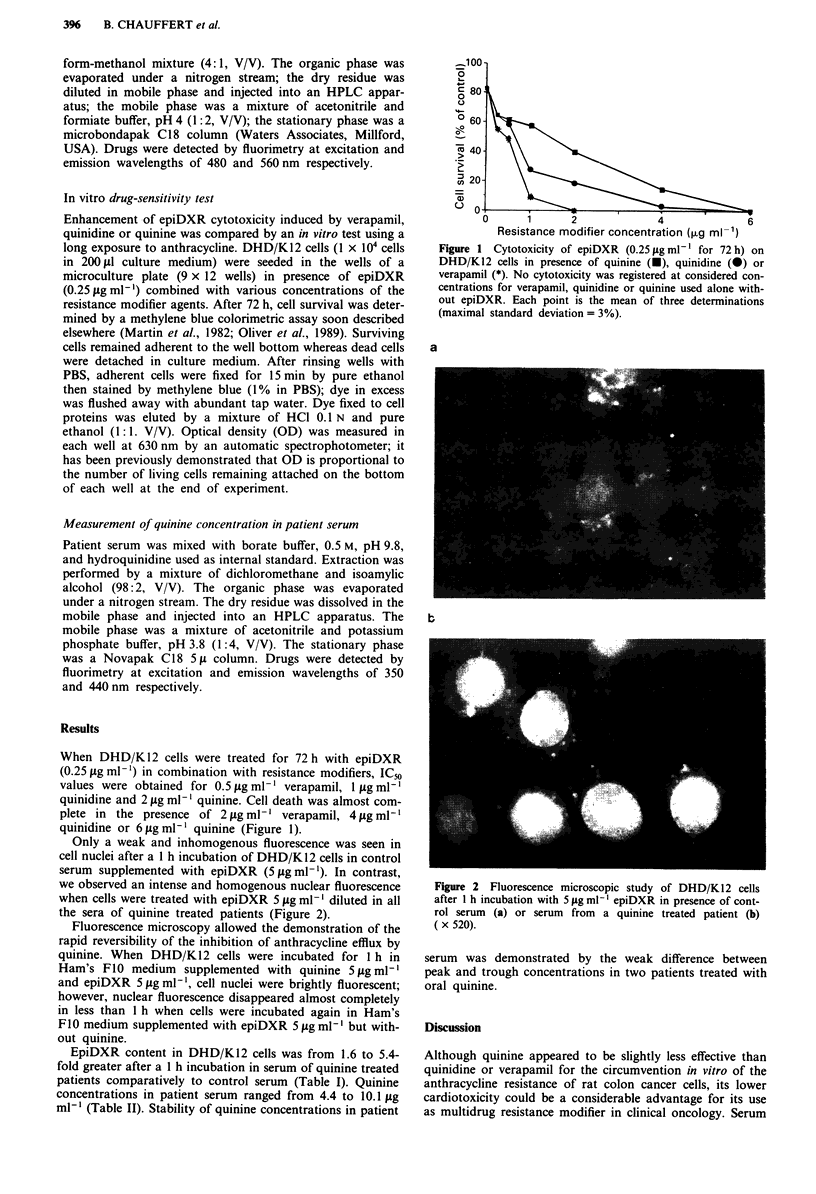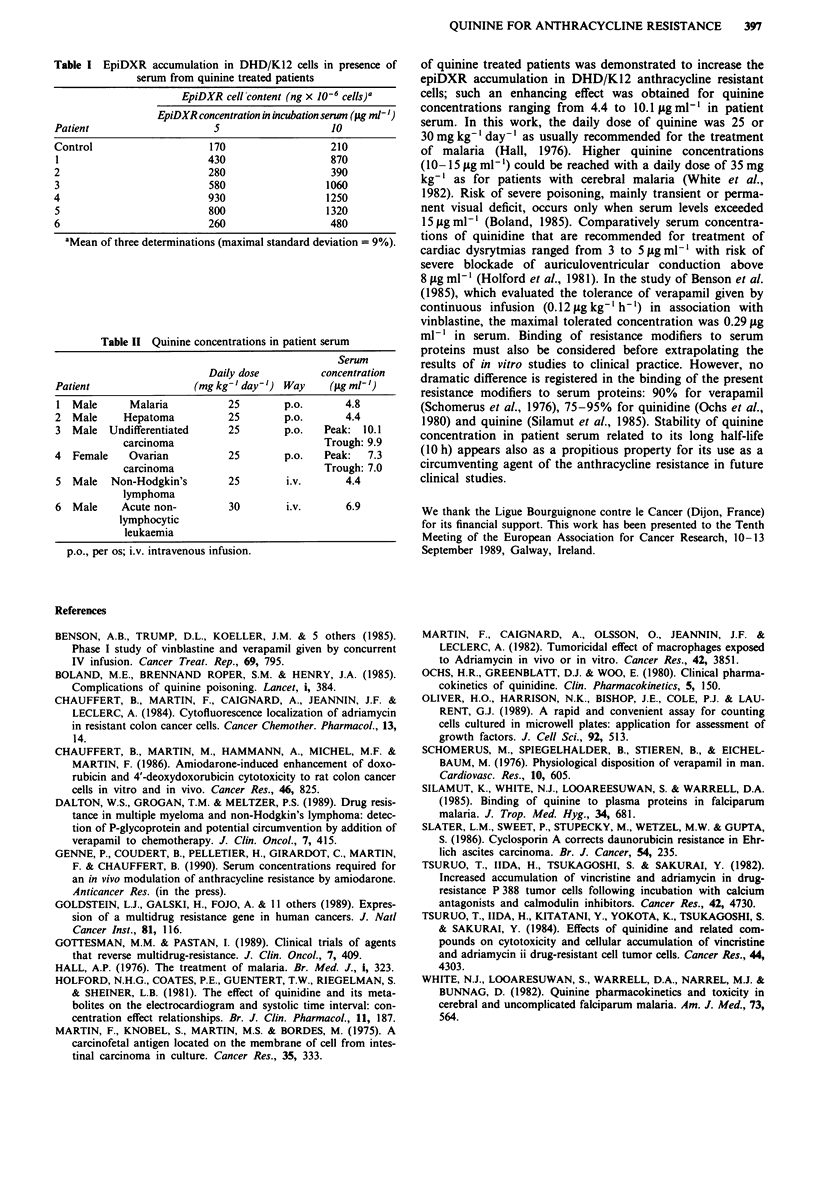# Potential usefulness of quinine to circumvent the anthracycline resistance in clinical practice.

**DOI:** 10.1038/bjc.1990.305

**Published:** 1990-09

**Authors:** B. Chauffert, H. Pelletier, C. Corda, E. Solary, L. Bedenne, D. Caillot, F. Martin

**Affiliations:** Research Group on Digestive Cancers, INSERM U.252, Dijon, France.

## Abstract

**Images:**


Br. J. Cancer (1990), 62, 395-397 © Macmillan Press Ltd., 1990

### Potential usefulness of quinine to circumvent the anthracycline resistance in clinical practice

B. Chauffert[1], H. Pelletier[1], C. Corda[2], E. Solary[3], L. Bedenne[4], D. Caillot[3] & F. Martin[1]

[1]*Research Group on Digestive Cancers, INSERM U.252;* [2]*Laboratory of Pharmacology;* [3]*Department of Haematology; and* [4]*Department of Hepato-gastroenterology, Faculty of Medicine and University Hospital, Dijon, France.*

**Summary** Quinine, the widely used antimalaria agent, was found to increase the cytotoxicity of epidoxorubicin (epiDXR) in resistant DHD/K12 rat colon cancer cells *in vitro*. Quinine appeared as slightly less effective than quinidine or verapamil for anthracycline potentiation but its weaker cardiotoxicity could counterbalance this disadvantage *in vivo*. Serum from six patients treated by conventional doses of quinine (25–30 mg $kg^{-1}$ $day^{-1}$) was demonstrated to enhance the accumulation of epiDXR in DHD/K12 cells as judged by fluorescence microscopy and HPLC assay (1.6 to 6-fold compared with control serum). In this patients quinine concentrations in serum ranged from 4.4 to 10.1 $\mu g\,ml^{-1}$. Our results suggest that quinine could be safely used as anthracycline resistance modifier in clinical practice.

Primary or acquired resistance to anthracyclines of human cancers is partly associated with the overexpression of a membrane glycoprotein (P 170) that effluxes drugs out of cancer cells (Goldstein *et al.*, 1989; Dalton *et al.*, 1989). Anthracycline resistance may be altered *in vitro* by a variety of agents such as verapamil (Tsuruo *et al.*, 1982), quinidine (Tsuruo *et al.*, 1984), amiodarone (Chauffert *et al.*, 1986) or cyclosporine (Slater *et al.*, 1986). However, use of resistance modifiers in clinical practice is still a problem due to the toxicity of these agents that precludes the achievement of effective concentrations in patient serum (Gottesman & Pastan, 1989; Genne *et al.*, 1990).

In this paper we report that quinine, the widely used antimalaria drug, enhanced *in vitro* the cytotoxicity of epidoxorubicin in resistant colon cancer cells. Moreover, serum of quinine treated patients was demonstrated to increase the cellular accumulation of the anthracycline in resistant cells.

### Materials and methods

### *Patients*

The first patient (no. 1) was treated for a chloroquine-resistant malaria; the other patients (nos 2–6) were treated after informed consent with a combination of quinine and doxorubicin for anthracycline resistant tumours. Quinine was given either per os or intravenously at a daily dose ordinarily used for malaria treatment (24–30 mg $kg^{-1}$ $day^{-1}$). When given per os, the daily dose of quinine was supplied in three regular intakes. Intravenous treatment was given as continuous infusion. Patient serum was collected at steady state 48 h at least after starting quinine administration. Peak and trough plasma concentrations of quinine were determined in two patients 2 and 8 h respectively after an oral intake. Serum of one of the authors was used as control. After blood collection, serum was centrifugated and stored at −80°C until assay.

### *Cancer cells*

The DHD/K12 cancer cell line was established in our laboratory from a chemically induced colon cancer in syngeneic BDIX rats (Martin *et al.*, 1975). Inherent resistance of DHD/K12 cells to anthracyclines is partly related to a drug efflux mechanism (Chauffert *et al.*, 1984) which is efficiently inhibited by amiodarone or verapamil (Chauffert *et al.*, 1986). Cells were grown as a monolayer adherent to the surface of culture flasks. Culture medium was a mixture of Ham's F10 medium and fetal bovine serum (10:1; V/V). For experiments, cells were detached from the culture flasks by a 10 min treatment with EDTA (0.2 mg $ml^{-1}$) and tryspin (2.5 mg $ml^{-1}$) in Hank's medium without $Ca^{2+}$ or $Mg^{2+}$.

### *Drugs*

Epidoxorubicin (epiDXR) was obtained from Farmitalia Carlo Erba laboratories (Milan, Italy). EpiDXR was preferred to doxorubicin because of its greater penetration and cytotoxicity *in vitro* in DHD/K12 cells. Daunorubicin used as internal standard for HPLC assay was purchased by Roger Bellon laboratories (Neuilly, France). Quinine sulphate was used for oral treatment and was obtained from Coopération Pharmacologique Française (Melun, France). Quinine formiate was used for intravenous treatment and was obtained from Vaillant-Defresne laboratories (Quinoforme, Paris, France). Quinine hydrochloride, quinidine hydrochloride and hydroquinidine hydrochloride used for *in vitro* experiments were obtained from Sigma (La Verpilliere, France). Verapamil hydrochloride was purchased from Biosedra laboratories (Malakoff, France).

### *Cytofluorescence study*

The intracellular accumulation of epiDXR in DHD/K12 cells was studied by UV illumination which induced a yellow-orange fluorescence at the intracellular sites of anthracycline localisation. We previously reported that low accumulation of anthracyclines in nucleus of primary resistant DHD/K12 cells was related to a drug efflux mechanism (Chauffert *et al.*, 1984). In the presence of sufficient concentration of a drug efflux inhibitor in incubation medium, anthracycline accumulation increased in cancer cells and then a bright fluorescence was observed in nuclei. For microscopic examination, DHD/ K12 cells were cultivated for 24 h on glass coverslips then exposed for 1 h to epiDXR (5 $\mu g\,ml^{-1}$) diluted in patient or control serum. After rinsing with cold phosphate buffered saline (PBS), cells were examined under an UV fluorescence microscope (Leitz, Weitzlar, FR Germany).

### *EpiDXR uptake in cancer cells*

DHD/K12 cells in suspension were incubated for 1 h at 37°C with epiDXR (5 or 10 $\mu g\,ml^{-1}$) diluted in patient or control serum. After rinsing twice with cold PBS and centrifugation, cells pellets were mixed with daunorubicin diluted in borate buffer, pH 9.4. Anthracyclines were extracted by a chloro-

Correspondence: B. Chauffert, INSERM U.252, Faculty of Medicine, 7 boulevard Jeanne d'Arc, F-21033 Dijon Cedex, France.
Received 3 November 1989; and in revised form 19 March 1990.

form-methanol mixture (4:1, V/V). The organic phase was evaporated under a nitrogen stream; the dry residue was diluted in mobile phase and injected into an HPLC apparatus; the mobile phase was a mixture of acetonitrile and formiate buffer, pH 4 (1:2, V/V); the stationary phase was a microbondapak C18 column (Waters Associates, Millford, USA). Drugs were detected by fluorimetry at excitation and emission wavelengths of 480 and 560 nm respectively.

### In vitro *drug-sensitivity test*

Enhancement of epiDXR cytotoxicity induced by verapamil, quinidine or quinine was compared by an *in vitro* test using a long exposure to anthracycline. DHD/K12 cells ($1 \times 10^4$ cells in 200 μl culture medium) were seeded in the wells of a microculture plate (9 × 12 wells) in presence of epiDXR (0.25 μg $ml^{-1}$) combined with various concentrations of the resistance modifier agents. After 72 h, cell survival was determined by a methylene blue colorimetric assay soon described elsewhere (Martin *et al.*, 1982; Oliver *et al.*, 1989). Surviving cells remained adherent to the well bottom whereas dead cells were detached in culture medium. After rinsing wells with PBS, adherent cells were fixed for 15 min by pure ethanol then stained by methylene blue (1% in PBS); dye in excess was flushed away with abundant tap water. Dye fixed to cell proteins was eluted by a mixture of HCl 0.1 N and pure ethanol (1:1. V/V). Optical density (OD) was measured in each well at 630 nm by an automatic spectrophotometer; it has been previously demonstrated that OD is proportional to the number of living cells remaining attached on the bottom of each well at the end of experiment.

### *Measurement of quinine concentration in patient serum*

Patient serum was mixed with borate buffer, 0.5 M, pH 9.8, and hydroquinidine used as internal standard. Extraction was performed by a mixture of dichloromethane and isoamylic alcohol (98:2, V/V). The organic phase was evaporated under a nitrogen stream. The dry residue was dissolved in the mobile phase and injected into an HPLC apparatus. The mobile phase was a mixture of acetonitrile and potassium phosphate buffer, pH 3.8 (1:4, V/V). The stationary phase was a Novapak C18 5 μ column. Drugs were detected by fluorimetry at excitation and emission wavelengths of 350 and 440 nm respectively.

### Results

When DHD/K12 cells were treated for 72 h with epiDXR (0.25 μg $ml^{-1}$) in combination with resistance modifiers, $IC_{50}$ values were obtained for 0.5 μg $ml^{-1}$ verapamil, 1 μg $ml^{-1}$ quinidine and 2 μg $ml^{-1}$ quinine. Cell death was almost complete in the presence of 2 μg $ml^{-1}$ verapamil, 4 μg $ml^{-1}$ quinidine or 6 μg $ml^{-1}$ quinine (Figure 1).

Only a weak and inhomogenous fluorescence was seen in cell nuclei after a 1 h incubation of DHD/K12 cells in control serum supplemented with epiDXR (5 μg $ml^{-1}$). In contrast, we observed an intense and homogenous nuclear fluorescence when cells were treated with epiDXR 5 μg $ml^{-1}$ diluted in all the sera of quinine treated patients (Figure 2).

Fluorescence microscopy allowed the demonstration of the rapid reversibility of the inhibition of anthracycline efflux by quinine. When DHD/K12 cells were incubated for 1 h in Ham's F10 medium supplemented with quinine 5 μg $ml^{-1}$ and epiDXR 5 μg $ml^{-1}$, cell nuclei were brightly fluorescent; however, nuclear fluorescence disappeared almost completely in less than 1 h when cells were incubated again in Ham's F10 medium supplemented with epiDXR 5 μg $ml^{-1}$ but without quinine.

EpiDXR content in DHD/K12 cells was from 1.6 to 5.4-fold greater after a 1 h incubation in serum of quinine treated patients comparatively to control serum (Table I). Quinine concentrations in patient serum ranged from 4.4 to 10.1 μg $ml^{-1}$ (Table II). Stability of quinine concentrations in patient

**Figure 1** Cytotoxicity of epiDXR (0.25 μg $ml^{-1}$ for 72 h) on DHD/K12 cells in presence of quinine (■), quinidine (●) or verapamil (*). No cytotoxicity was registered at considered concentrations for verapamil, quinidine or quinine used alone without epiDXR. Each point is the mean of three determinations (maximal standard deviation = 3%).

**Figure 2** Fluorescence microscopic study of DHD/K12 cells after 1 h incubation with 5 μg $ml^{-1}$ epiDXR in presence of control serum (**a**) or serum from a quinine treated patient (**b**) (× 520).

serum was demonstrated by the weak difference between peak and trough concentrations in two patients treated with oral quinine.

### Discussion

Although quinine appeared to be slightly less effective than quinidine or verapamil for the circumvention *in vitro* of the anthracycline resistance of rat colon cancer cells, its lower cardiotoxicity could be a considerable advantage for its use as multidrug resistance modifier in clinical oncology. Serum

**Table I** EpiDXR accumulation in DHD/K12 cells in presence of serum from quinine treated patients

| | *EpiDXR cell content (ng × $10^{-6}$ cells)*[a] | |
|---|---|---|
| | *EpiDXR concentration in incubation serum (μg $ml^{-1}$)* | |
| *Patient* | *5* | *10* |
| Control | 170 | 210 |
| 1 | 430 | 870 |
| 2 | 280 | 390 |
| 3 | 580 | 1060 |
| 4 | 930 | 1250 |
| 5 | 800 | 1320 |
| 6 | 260 | 480 |

[a]Mean of three determinations (maximal standard deviation = 9%).

**Table II** Quinine concentrations in patient serum

| *Patient* | | | *Daily dose (mg $kg^{-1}$ $day^{-1}$)* | *Way* | *Serum concentration (μg $ml^{-1}$)* |
|---|---|---|---|---|---|
| 1 | Male | Malaria | 25 | p.o. | 4.8 |
| 2 | Male | Hepatoma | 25 | p.o. | 4.4 |
| 3 | Male | Undifferentiated carcinoma | 25 | p.o. | Peak: 10.1<br>Trough: 9.9 |
| 4 | Female | Ovarian carcinoma | 25 | p.o. | Peak: 7.3<br>Trough: 7.0 |
| 5 | Male | Non-Hodgkin's lymphoma | 25 | i.v. | 4.4 |
| 6 | Male | Acute non-lymphocytic leukaemia | 30 | i.v. | 6.9 |

p.o., per os; i.v. intravenous infusion.

of quinine treated patients was demonstrated to increase the epiDXR accumulation in DHD/K12 anthracycline resistant cells; such an enhancing effect was obtained for quinine concentrations ranging from 4.4 to 10.1 μg $ml^{-1}$ in patient serum. In this work, the daily dose of quinine was 25 or 30 mg $kg^{-1}$ $day^{-1}$ as usually recommended for the treatment of malaria (Hall, 1976). Higher quinine concentrations (10–15 μg $ml^{-1}$) could be reached with a daily dose of 35 mg $kg^{-1}$ as for patients with cerebral malaria (White *et al.*, 1982). Risk of severe poisoning, mainly transient or permanent visual deficit, occurs only when serum levels exceeded 15 μg $ml^{-1}$ (Boland, 1985). Comparatively serum concentrations of quinidine that are recommended for treatment of cardiac dysrytmias ranged from 3 to 5 μg $ml^{-1}$ with risk of severe blockade of auriculoventricular conduction above 8 μg $ml^{-1}$ (Holford *et al.*, 1981). In the study of Benson *et al.* (1985), which evaluated the tolerance of verapamil given by continuous infusion (0.12 μg $kg^{-1}$ $h^{-1}$) in association with vinblastine, the maximal tolerated concentration was 0.29 μg $ml^{-1}$ in serum. Binding of resistance modifiers to serum proteins must also be considered before extrapolating the results of *in vitro* studies to clinical practice. However, no dramatic difference is registered in the binding of the present resistance modifiers to serum proteins: 90% for verapamil (Schomerus *et al.*, 1976), 75–95% for quinidine (Ochs *et al.*, 1980) and quinine (Silamut *et al.*, 1985). Stability of quinine concentration in patient serum related to its long half-life (10 h) appears also as a propitious property for its use as a circumventing agent of the anthracycline resistance in future clinical studies.

We thank the Ligue Bourguignone contre le Cancer (Dijon, France) for its financial support. This work has been presented to the Tenth Meeting of the European Association for Cancer Research, 10–13 September 1989, Galway, Ireland.

### References

BENSON, A.B., TRUMP, D.L., KOELLER, J.M. & 5 others (1985). Phase I study of vinblastine and verapamil given by concurrent IV infusion. *Cancer Treat. Rep.*, **69**, 795.

BOLAND, M.E., BRENNAND ROPER, S.M. & HENRY, J.A. (1985). Complications of quinine poisoning. *Lancet*, **i**, 384.

CHAUFFERT, B., MARTIN, F., CAIGNARD, A., JEANNIN, J.F. & LECLERC, A. (1984). Cytofluorescence localization of adriamycin in resistant colon cancer cells. *Cancer Chemother. Pharmacol.*, **13**, 14.

CHAUFFERT, B., MARTIN, M., HAMMANN, A., MICHEL, M.F. & MARTIN, F. (1986). Amiodarone-induced enhancement of doxorubicin and 4'-deoxydoxorubicin cytotoxicity to rat colon cancer cells in vitro and in vivo. *Cancer Res.*, **46**, 825.

DALTON, W.S., GROGAN, T.M. & MELTZER, P.S. (1989). Drug resistance in multiple myeloma and non-Hodgkin's lymphoma: detection of P-glycoprotein and potential circumvention by addition of verapamil to chemotherapy. *J. Clin. Oncol.*, **7**, 415.

GENNE, P., COUDERT, B., PELLETIER, H., GIRARDOT, C., MARTIN, F. & CHAUFFERT, B. (1990). Serum concentrations required for an *in vivo* modulation of anthracycline resistance by amiodarone. *Anticancer Res.* (in the press).

GOLDSTEIN, L.J., GALSKI, H., FOJO, A. & 11 others (1989). Expression of a multidrug resistance gene in human cancers. *J. Natl Cancer Inst.*, **81**, 116.

GOTTESMAN, M.M. & PASTAN, I. (1989). Clinical trials of agents that reverse multidrug-resistance. *J. Clin. Oncol.*, **7**, 409.

HALL, A.P. (1976). The treatment of malaria. *Br. Med. J.*, **i**, 323.

HOLFORD, N.H.G., COATES, P.E., GUENTERT, T.W., RIEGELMAN, S. & SHEINER, L.B. (1981). The effect of quinidine and its metabolites on the electrocardiogram and systolic time interval: concentration effect relationships. *Br. J. Clin. Pharmacol.*, **11**, 187.

MARTIN, F., KNOBEL, S., MARTIN, M.S. & BORDES, M. (1975). A carcinofetal antigen located on the membrane of cell from intestinal carcinoma in culture. *Cancer Res.*, **35**, 333.

MARTIN, F., CAIGNARD, A., OLSSON, O., JEANNIN, J.F. & LECLERC, A. (1982). Tumoricidal effect of macrophages exposed to Adriamycin in vivo or in vitro. *Cancer Res.*, **42**, 3851.

OCHS, H.R., GREENBLATT, D.J. & WOO, E. (1980). Clinical pharmacokinetics of quinidine. *Clin. Pharmacokinetics*, **5**, 150.

OLIVER, H.O., HARRISON, N.K., BISHOP, J.E., COLE, P.J. & LAURENT, G.J. (1989). A rapid and convenient assay for counting cells cultured in microwell plates: application for assessment of growth factors. *J. Cell Sci.*, **92**, 513.

SCHOMERUS, M., SPIEGELHALDER, B., STIEREN, B., & EICHELBAUM, M. (1976). Physiological disposition of verapamil in man. *Cardiovasc. Res.*, **10**, 605.

SILAMUT, K., WHITE, N.J., LOOAREESUWAN, S. & WARRELL, D.A. (1985). Binding of quinine to plasma proteins in falciparum malaria. *J. Trop. Med. Hyg.*, **34**, 681.

SLATER, L.M., SWEET, P., STUPECKY, M., WETZEL, M.W. & GUPTA, S. (1986). Cyclosporin A corrects daunorubicin resistance in Ehrlich ascites carcinoma. *Br. J. Cancer*, **54**, 235.

TSURUO, T., IIDA, H., TSUKAGOSHI, S. & SAKURAI, Y. (1982). Increased accumulation of vincristine and adriamycin in drug-resistance P 388 tumor cells following incubation with calcium antagonists and calmodulin inhibitors. *Cancer Res.*, **42**, 4730.

TSURUO, T., IIDA, H., KITATANI, Y., YOKOTA, K., TSUKAGOSHI, S. & SAKURAI, Y. (1984). Effects of quinidine and related compounds on cytotoxicity and cellular accumulation of vincristine and adriamycin ii drug-resistant cell tumor cells. *Cancer Res.*, **44**, 4303.

WHITE, N.J., LOOARESUWAN, S., WARRELL, D.A., NARREL, M.J. & BUNNAG, D. (1982). Quinine pharmacokinetics and toxicity in cerebral and uncomplicated falciparum malaria. *Am. J. Med.*, **73**, 564.